# Effects of jujube (*Ziziphus jujuba* mill.) fruit extracts on oxidative stress: A systematic review and meta‐analysis of rodent studies

**DOI:** 10.1002/fsn3.4234

**Published:** 2024-05-22

**Authors:** Di Zhu, Yu Zhu, Hao Tan, Rui Ding, Qiangqiang Dai, Xiaoming Du, Yulin Liu, Rensong Yue

**Affiliations:** ^1^ Hospital of Chengdu University of Traditional Chinese Medicine Chengdu China; ^2^ Chengdu Sport University Chengdu China

**Keywords:** animal model, antioxidant, jujube, meta‐analysis, systematic review

## Abstract

This study aimed to evaluate the effects of jujube (*Ziziphus jujuba* Mill.) fruit extracts on oxidative stress levels in rodent models. Animal studies meeting the inclusion criteria were retrieved from PubMed, Web of Science, Embase, China National Knowledge Infrastructure (CNKI), Wanfang Data Knowledge Service Platform, and VIP Periodical Service Platform. The Systematic Review Center for Laboratory Animal Experimentation (SYRCLE) risk‐of‐bias tool was used to evaluate the risk of bias in the included studies. A meta‐analysis was performed based on the guidelines provided in the Cochrane Handbook for Systematic Reviews of Interventions (CHSRI) by using Stata 17.0 software. Nineteen studies were included in the meta‐analysis. Jujube fruit extracts significantly decreased the level of malonaldehyde (MDA) and increased the levels of superoxide dismutase (SOD) and glutathione peroxidase (GSH‐Px). Meanwhile, there was no significant improvement in the catalase (CAT) levels. In addition, there was considerable heterogeneity in the results of the meta‐analysis. The results of the subgroup analysis indicated that the animal model, type of extracts, and source of target parameters may have contributed to the heterogeneity. Jujube fruit extracts are healthy and effective antioxidant dietary supplements that may be an effective adjunctive therapy for diseases in which oxidative stress is a major pathological factor. However, the overall methodological quality of the included studies was low, and additional research is warranted.

## Graphical abstract

This study aimed to evaluate the effects of jujube (*Ziziphus jujuba* Mill.) fruit extracts on oxidative stress levels in rodent models. The results of this systematic review and meta‐analysis suggest that jujube fruit extracts are healthy and effective antioxidant dietary supplements that may be an effective adjunctive therapy for diseases in which oxidative stress is a major pathological factor.

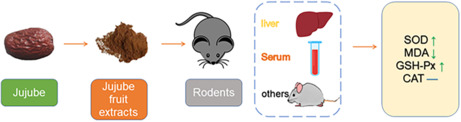

## 1 INTRODUCTION

Oxidative stress refers to an imbalance in the levels of oxidants and antioxidants in favor of the oxidants. This imbalance can cause molecular damage and disruption in redox signaling and control (Sies, 2015). Reactive derivatives of oxygen, such as superoxide (O_2_^·−^), hydrogen peroxide (H_2_O_2_), hydroxyl radical (^·^OH), ozone, and singlet oxygen are all reactive oxygen species (ROS) (Forman & Zhang, 2021; Powers et al., 2020). Although the creation of ROS is a healthy and regulated process, excessive ROS production in cells may lead to redox disturbances that cause oxidative damage to cellular components. Numerous disorders, including chronic fatigue syndrome, liver disease, metabolic syndrome, diabetes, and hyperlipidemia, among others, are considerably influenced by oxidative stress (Le Lay et al., 2014; Masenga et al., 2023; McGill & Hinson, 2020; Morris et al., 2019; Seen, 2021; Unsal et al., 2021). In these diseases, oxidative stress, as a primary cause of pathology or the secondary contributor to disease progression, is a key therapeutic target that requires attention (Forman & Zhang, 2021). Enzymatic and nonenzymatic antioxidant systems, which collectively create an efficient in vivo antioxidant defense system, are the two primary antioxidant systems. Enzymatic antioxidants primarily include superoxide dismutase (SOD), catalase (CAT), glutathione peroxidase (GSH‐Px), and glutathione reductase (GR). These enzymes can effectively resist oxidative damage. Nonenzymatic antioxidants, such as vitamins C and E, can also directly protect against oxidative stress and collaborate with endogenous enzymatic antioxidants. This helps scavenge ROS and boost antioxidant activity more effectively (Jiao et al., 2016).

Oxidative stress, a component of several diseases, has drawn more attention in recent years. However, the development of therapeutic antioxidant approaches has not been very successful. Researchers are exploring various antioxidant treatment strategies, and supplementation with dietary antioxidants is one of them (Forman & Zhang, 2021). Synthetic antioxidants like butylated hydroxyanisole and butylated hydroxytoluene have been employed in the food sector. However, these antioxidants have been linked to cancer and liver damage (Arunachalam et al., 2022). These developments have led to an increased interest in naturally occurring antioxidants derived from plants. Numerous herbs are considered safe and healthy, and their active components can serve as both enzymatic and nonenzymatic antioxidants.

**FIGURE 1:**
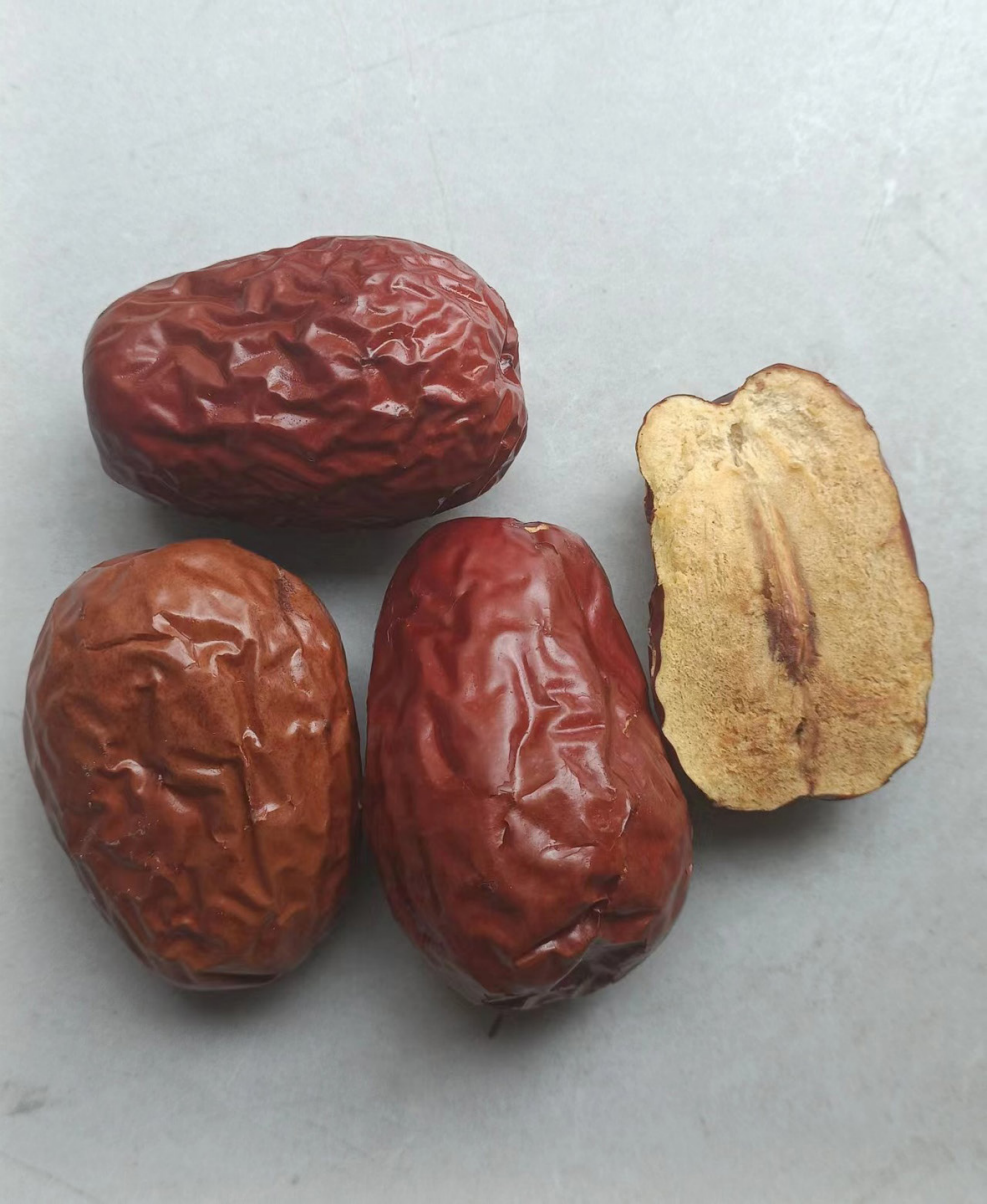
Jujube (*Ziziphus jujuba* Mill.) fruit.

Jujube (*Ziziphus jujuba* Mill.), also known as Chinese date, belongs to the genus *Ziziphus* of the family Rhamnaceae and is native to China. Of the 170 *Ziziphus* species, it is the most significant in terms of both ecology and commerce, and it also occupies the greatest cultivated area (Li, Muhammad, et al., 2023; Li, Pan, et al., 2023). Jujube fruit (Figure 1), which has been used in China for more than 4000 years as a food supplement and traditional herbal medicine, is nutritious, has multiple health benefits, and is regarded as an extremely valuable fruit and excellent medicinal herb that can prolong life by promoting enhanced digestion, better sleep, and blood nourishment (Chen & Tsim, 2020). Among the 8000 odd traditional Chinese medicine (TCM) prescriptions included in the Chinese Medicated Diet Dictionary, jujube fruit appears 400 times, making it the most used TCM herb (Zhu, 2008). Jujube fruit contains various nutrients, including polysaccharides, polyphenols, amino acids, triterpenic acids, fatty acids, nucleotides, dietary fiber, alkaloids, vitamins, and other nutrients. It has antioxidant, anti‐inflammatory, anticancer, antihyperglycemic, antihyperlipidemic, immune regulatory, neuroprotective, sedative, and antiviral properties (Lu et al., 2021). Most of the reported therapeutic effects are primarily attributed to the antioxidant and anti‐inflammatory properties of jujube fruit extracts, especially the antioxidant properties. The extracts act as enzymatic and nonenzymatic antioxidants and effectively inhibit ROS production and lipid peroxidation (Hong et al., 2020). Previous studies have shown that jujube fruit extracts may inhibit NF‐κB to suppress the expression of inflammatory proteins and activate Nrf2‐mediated antioxidant responses to ameliorate tissue damage (Huang et al., 2017; Kim et al., 2020).

Jujube fruit extracts have been shown to attenuate oxidative stress in several studies. However, systematic summaries of pertinent data are still lacking. Here, we conducted a systematic review and meta‐analysis of rodent experiments, for the first time, to evaluate the effects of jujube extracts on oxidative stress in rodent models, and provide a reference and evidence for the clinical application of jujube extracts as a dietary supplement to improve the outcomes of oxidative stress‐related diseases.

## 2 MATERIALS AND METHODS

The current systematic review and meta‐analysis were designed and carried out following the Preferred Reporting Items for Systematic Reviews and Meta‐Analysis (PRISMA). It was registered with PROSPERO (registration number: CRD42023426300).

### 2.1 Search strategy

The databases of PubMed, Web of Science, Embase, China National Knowledge Infrastructure (CNKI), Wanfang Data Knowledge Service Platform, and VIP Periodical Service Platform were searched from database inception until April 2023. The language was limited to English or Chinese. Medical subject headings (MeSH) and free words for database searches were as follows: [(“Ziziphus” OR “Jujube” OR “Chinese jujube” OR “*Ziziphus jujuba* Mill” OR “jujube polysaccharide”) AND (“oxidative stress” OR “antioxidant” OR “superoxide dismutase” OR “glutathione” OR “glutathione peroxidase” OR “malondialdehyde” OR “catalase”) AND (“rat” OR “mice” OR “mouse” OR “animal” OR “rodent” OR “murinae”)]. The specific retrieval strategies are listed in Table S1.

### 2.2 Inclusion criteria

(1) Model: studies used rodent models; (2) intervention: jujube fruit extracts with all dosage and duration; (3) comparison: the control group was untreated controlled or vehicle controlled; and (4) outcomes: the outcome parameters included at least one of SOD, MDA, GSH‐Px, and CAT.

### 2.3 Exclusion criteria

(1) Studies not in English or Chinese; (2) in vitro studies or clinical trials; (3) nonjujube fruit extracts; (4) free diet with the extracts; (5) combined with other therapies; (6) reviews, cross‐over studies, and studies without a separate control group; (7) duplicate publication; and (8) data or full text of the studies was not available.

### 2.4 Data extraction

After importing all the retrieved literature into EndNote X9, duplicates were removed. Two independent researchers conducted preliminary screening based on title and abstract to exclude irrelevant literature, and the rest were reviewed by obtaining the complete text to further weed out literature that did not match the inclusion criteria. Disputes regarding the inclusion of a study were settled by discussing with a third researcher. Details were extracted from selected studies: (1) the first author and publication year; (2) characteristics of experimental animals, including animal species, sex, sample size, and weight; (3) the models and modeling methods; (4) information regarding intervention and control groups (administration, dosage, and duration of intervention); and (5) outcome indicator and sources of target parameter. For studies requiring data extraction from images, we used GetData Graph Digitizer software (version 2.26) to extract the corresponding outcome indicators. To categorize therapeutic drugs into subgroups based on data from each original study, we used the Cochrane Handbook for Systematic Reviews of Interventions (CHSRI) and grouped the findings from various subgroups into a single treatment group for analysis. When extracting data of the identical outcome indicator obtained from multiple test samples, we used the mean value.

### 2.5 Quality assessment

The quality of the included studies was evaluated by two researchers independently using the Systematic Review Center for Laboratory Animal Experimentation (SYRCLE) risk‐of‐bias tool. The assessment contains 10 entries: (1) sequence generation, (2) baseline characteristics, (3) allocation concealment, (4) random housing, (5) blinding (for animal breeders and researchers), (6) random outcome assessment, (7) blinding (for outcome evaluator), (8) incomplete outcome data, (9) selective outcome reporting, and (10) other sources of bias. Each entry was identified as “low risk,” “high risk,” or “unclear risk”. Any disagreement that arose from the evaluation was settled by discussing it with a third researcher.

### 2.6 Data synthesis and analysis

The STATA software version 17.0 was used to conduct the statistical analysis. Standardized mean difference (SMD) and 95% confidence interval (95% CI) were used to assess continuous outcomes. *p* < .05 was considered statistically significant. Then, a general classification of effect sizes into small, medium, and large was made using SMD cutoff points of 0.2, 0.5, and 0.8. Random effects models (DerSimonian–Laird) were used to calculate the combined results. Statistical heterogeneity was assessed using I‐squared (*I* ^2^), with *I* ^2^ > 50% indicating significant heterogeneity. Subgroup analysis was performed to assess the sources of interstudy heterogeneity, in which we considered variables including animal model, type of extracts, duration, and source of target parameters. Sensitivity analysis was carried out to assess the overall results' stability. Publication bias was evaluated with the Egger's test if there were at least 10 studies for each outcome.

## 3 RESULTS

### 3.1 Study selection

**FIGURE 2:**
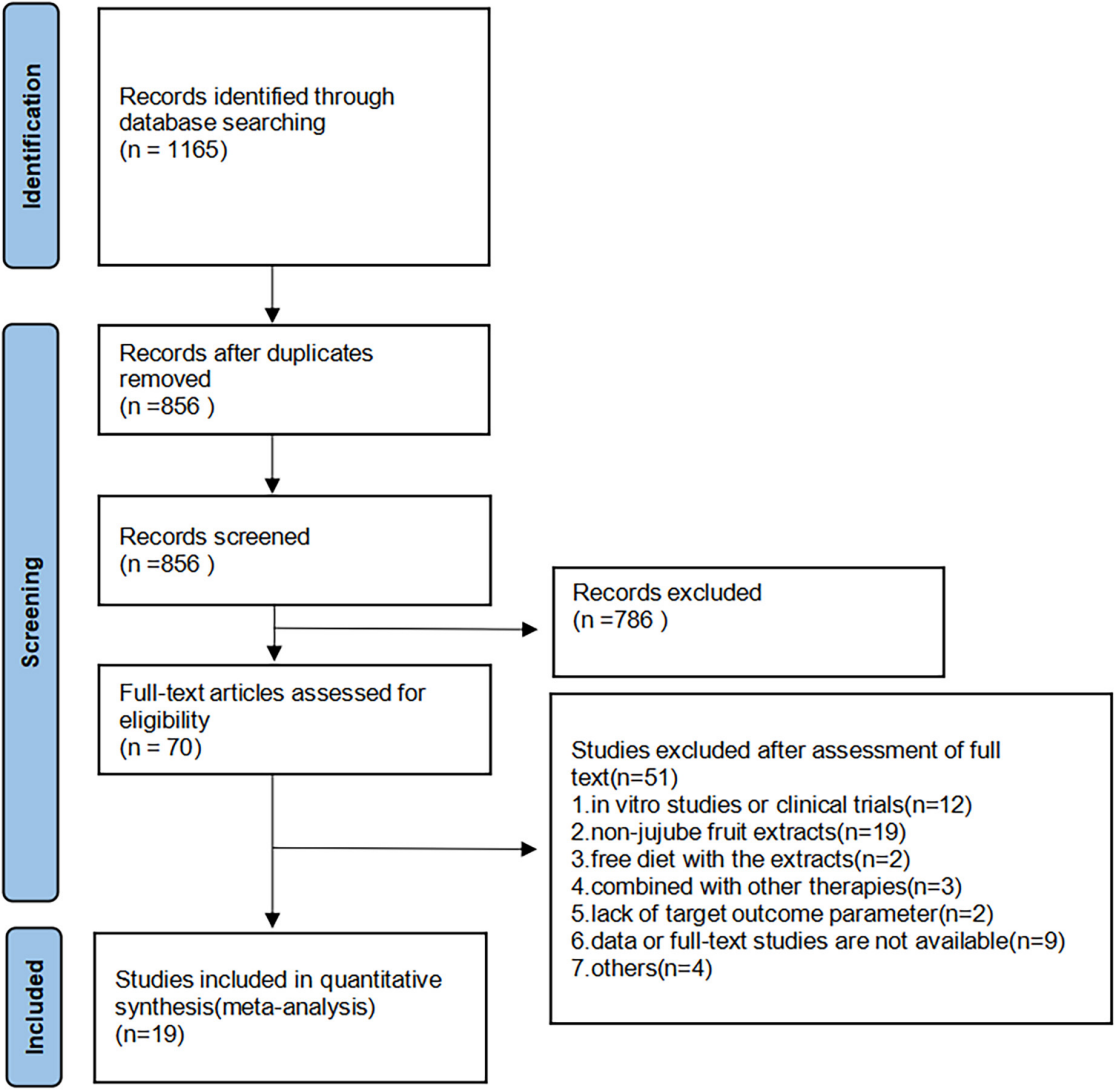
Flowchart for selection of studies.

A total of 1165 studies were retrieved from six databases (143 from PubMed, 112 from Web of Science, 307 from Embase, 262 from CNKI, 233 from Wanfang, and 108 from VIP). After duplicates were removed, 856 studies remained. After reading the title and abstract, we found 70 of these to be eligible for full‐text screening. Nineteen studies were eventually included in the systematic review and meta‐analysis after 51 studies that did not match the inclusion criteria were removed (Cai et al., 2018; Chi et al., 2015; Du & Liu, 2008; Feng et al., 2019; Gu et al., 2006, 2012; Huang et al., 2017; Kang & Li, 2010; Li et al., 2005; Liu et al., 2015, 2017; Mohebbati et al., 2021; Resim et al., 2020; Shao & Tang, 2015; Shen et al., 2009; Sheng, 2004; Wang, 2010; Wang et al., 2012; Xie et al., 2018). A detailed flow chart of the process is presented in Figure 2.

### 3.2 Study characteristics

The 19 studies included were published between 2004 and 2021. In these studies, the animal models were either rats or mice. Sprague–Dawley rats (SD rats) were used in four studies (Chi et al., 2015; Gu et al., 2012; Resim et al., 2020; Shao & Tang, 2015), Wistar rats were used in one study (Mohebbati et al., 2021), Kunming mice were used in six studies (Cai et al., 2018; Du & Liu, 2008; Gu et al., 2006; Kang & Li, 2010; Liu et al., 2015; Wang et al., 2012), Balb/c mice were used in two studies (Huang et al., 2017; Liu et al., 2017), and ICR mice were used in six studies (Feng et al., 2019; Li et al., 2005; Shen et al., 2009; Sheng, 2004; Wang, 2010; Xie et al., 2018). Among animal models, a chronic fatigue syndrome model was used in two studies (Chi et al., 2015; Shao & Tang, 2015), a toxic liver injury model was established in five studies (Gu et al., 2006; Huang et al., 2017; Liu et al., 2015; Shen et al., 2009; Wang et al., 2012), an alcoholic liver model was established in three studies (Cai et al., 2018; Gu et al., 2012; Liu et al., 2017), a cavernous nerve injury model and a sham surgery model were collectively established in one study (Resim et al., 2020), a diabetes model was established in two studies (Feng et al., 2019; Xie et al., 2018), a hyperlipidemia model was established in one study (Kang & Li, 2010), an exercise‐induced fatigue model was established in two studies (Du & Liu, 2008; Wang, 2010), and a testicular toxicity model was established in one study (Mohebbati et al., 2021). Two studies did not establish the model (Li et al., 2005; Sheng, 2004). All intervention groups were treated with jujube fruit extracts, of which water extracts of jujube fruits were used in 11 studies (Chi et al., 2015; Feng et al., 2019; Gu et al., 2006, 2012; Li et al., 2005; Liu et al., 2017; Mohebbati et al., 2021; Resim et al., 2020; Shao & Tang, 2015; Wang, 2010; Wang et al., 2012; Xie et al., 2018), ethanol extracts of jujube fruits were used in seven studies (Cai et al., 2018; Huang et al., 2017; Kang & Li, 2010; Liu et al., 2015; Mohebbati et al., 2021; Shen et al., 2009; Sheng, 2004), and the extraction method was not mentioned in one study (Du & Liu, 2008). However, owing to the differences in the extraction processes and specific technology, the composition of the jujube fruit extracts obtained from the same macerated medium may have differed. The dosage, timing, and duration of intervention with jujube fruit extracts used in the different studies were different. Detailed characteristics of the included studies are listed in Table 1.

**TABLE 1:** Basic characteristics of included studies.

Study	Species (sex; n = intervention/control group), weight	Model	Intervention	Control	Administration/duration	Outcomes	Source of target parameters
Sheng (2004)	ICR mice (half male and half female; 30/10), 20–24 g	None	Ethanol extracts of jujube fruits (Jujube flavone); 0.125, 0.25, and 0.5 mL/day	NM	By SIJ, 28 days	SOD, MDA, and CAT	Serum, liver, and brain
Li et al. (2005)	ICR mice (NM; 30/10), NM	None	Water extracts of jujube fruits (jujube polysaccharide); 100, 200, and 400 mg/kg/day	Same volume of NS	By intragastric, 4 weeks	SOD, CAT, and MDA	Serum, liver, and brain
Gu et al. (2006)	Kunming mice (half male and half female; 30/10), 20–24 g	Acute hepatotoxicity model (10 mL·kg‐1 of 0.1% CCl_4_ emulsion was intraperitoneally injected for 5 days)	Water extracts of jujube fruits (jujube polysaccharide); 2, 6, and 10 g/kg/day	Equivalent tap water	By oral, 15 days (the intervention was carried out for 10 days before modeling and 5 days at the same time as modeling)	CAT, SOD, MDA, GSH‐Px, and liver pathology	Serum
Du and Liu (2008)(A)	Kunming mice (male; 20/10), 22–26 g	Exercise‐induced fatigue model (swim training for 4 weeks, 6 days a week, training time gradually increased). The mice were killed on the last day before exercise	Extracts of jujube fruits (crude jujube polysaccharide); 100 and 200 mg/kg/day	Same volume of NS	By intragastric, 4 weeks (the intervention was carried out simultaneously with modeling)	SOD, GSH‐Px, CK, LDH, GOT, GPT, and time to exhaustion	Serum
Du and Liu (2008)(B)	Kunming mice (male; 20/10), 22–26 g	Exercise‐induced fatigue model (swim training for 4 weeks, 6 days a week, training time gradually increased. On the last day, the mice were killed after swimming to exhaustion with 5% of their body weight in lead wire tied to their tails)	Extracts of jujube fruits (crude jujube polysaccharide); 100 and 200 mg/kg/day	Same volume of NS	By intragastric, 4 weeks (the intervention was carried out simultaneously with modeling)	SOD, GSH‐Px, CK, LDH, GOT, GPT, and time to exhaustion	Serum
Shen et al. (2009)	ICR mice (male; 20/10), 25–28 g	Hepatic injury model (CCl4 2 mL/kg/s (40%, v/v in olive oil) was administrated by subcutaneous injection)	Ethanol extracts of jujube fruits; 100 and 200 mg/kg/day	Distilled water	By intragastric, 10 days (the modeling was performed on the 8th day after the intervention)	SOD, CAT, GSH‐Px, GSH, MDA, ALT, AST, and liver pathology	Liver
Wang (2010)	ICR mice (male; 60/20), 18–22 g	Exercise‐induced fatigue model (swim training for 3 weeks, swim 30 min a day in the first week (no weight), then increase by 10 min a week thereafter (weight: hang 5% of the body weight of lead wire on the tail of the mouse))	Water extracts of jujube fruits (jujube polysaccharide); 100, 200, and 400 mg/kg/day	50 mL/kg/day of NS	By intragastric, 3 weeks (the intervention was carried out simultaneously with modeling)	SOD, GSH‐Px, CAT, MDA, body weight, time to exhaustion, CK, LDH, blood lactate, BUN, GPT, GOT, blood glucose, muscle glycogen, and liver glycogen	Heart, liver, spleen, and skeletal muscle
Kang and Li (2010)	Kunming mice (male; 10/10), 22–25 g	Hyperlipidemia model (0.2 mL /10 g high‐fat emulsion was administered intragastric for 14 days)	Ethanol extracts of jujube fruits; 1000 mg/kg/day	NM	By intragastric, 14 days (the intervention was carried out simultaneously with modeling)	SOD, MDA, TC, TG, LDL‐C, HDL‐C, and liver index	Serum
Wang et al. (2012)	Kunming mice (male; 30/10), 18–22 g	Acute hepatotoxicity model (0.8% CCl_4_/peanut oil mixture (v/v, 0.3 mL, ip))	Water extracts of jujube fruits (jujube polysaccharide); 100, 200, and 400 mg/kg/day	A single dose of NS (0.3 mL, ig)	By intragastric, 10 days (the intervention was performed before modeling)	MDA, GSH‐Px, SOD, body weight, liver weight, ALT, AST, and LDH	Liver
Gu et al. (2012)	SD rats (half male and half female; 30/10), 150–250 g	Alcoholic liver model (rats were given 56‐degree liquor (Beijing Erguotou) by intragastric administration (8 mL/kg) for 6 weeks)	Water extracts of jujube fruits (jujube polysaccharide); 4, 8, and 16 g/kg/day	Tap water	By intragastric, 6 weeks (the intervention was carried out simultaneously with modeling)	CAT, SOD, MDA, and GSH‐Px	Heart, liver, spleen, lung, and kidney
Shao and Tang (2015)	SD rats (male; 30/10), 180–220 g	Chronic fatigue syndrome model (electric shock, restraint stress, and cold water swim for 4 weeks)	Water extracts of jujube fruits (jujube polysaccharide); 100, 200, and 400 mg/kg/day	Same volume of NS	By intragastric, 4 weeks (the intervention was carried out simultaneously with modeling)	SOD, GSH‐Px, MDA, spleen index, thymus index, and splenic T lymphocyte transformation ability	Serum
Chi et al. (2015)	SD rats (NM; 30/10), 180–220 g	Chronic fatigue syndrome model (electric shock, restraint stress, and cold water swim)	Water extracts of jujube fruits (jujube polysaccharide conjugates); 100, 200, and 400 mg/kg/day	Same volume of NS	By intragastric, 30 days (the intervention was carried out simultaneously with modeling)	SOD, GSH‐Px, MDA, IL‐2, IL‐4, IL‐10, CD4, CD8, T cells proliferation, NK cells activity, and behavior test: MWM, OFT, and TST	Serum
Liu et al. (2015)(A)	Kunming mice (NM; 48/8), 25–28 g	Acute hepatic damage model (intraperitoneal injection of 0.4% CCl4 in peanut oil (v/v, 0.3 mL))	Ethanol extracts of jujube fruits (jujube polysaccharide); 100, 200, and 400 mg/kg	Same volume of the NS	By SIJ, one time (the intervention was performed 1, 3, or 6 h after modeling)	SOD, MDA, GSH‐Px, AST, ALT, LDH, and liver pathology	Liver
Liu et al. (2015)(B)	Kunming mice (NM; 48/8), 25–28 g	Acute hepatic damage model (intraperitoneal injection of 0.4% APAP (400 mg/kg body weight) in 0.5 mL physiological saline containing the appropriate amount of HPMC, PVP K30, and Lutrol F68 micro as common excipients)	Ethanol extracts of jujube fruits (jujube polysaccharide); 100, 200, and 400 mg/kg	Same volume of the NS	By SIJ, one time (the intervention was performed 1, 3, or 6 h after modeling)	SOD, MDA, GSH‐Px, AST, ALT, LDH, and liver pathology	Liver
Liu et al. (2017)(A)	Balb/c mice (NM; 18/6), 18–22 g	Acute alcohol‐induced liver damage model (alcohol (50%, v/v, 15 mL/kg) orally for a single dose each day for up to 7 days)	Water extracts of jujube fruits; 100, 200, and 400 mg/kg/day	Distilled water	By intragastric, 30 days (the intervention was performed before modeling)	SOD, MDA, GSH‐Px, body weight, liver index, TC, TG, ALT, AST, LDH, IL‐6, TNF‐α, iNOS, NF‐κB p65, liver pathology	Liver
Liu et al. (2017)(B)	Balb/c mice (NM; 18/6), 18–22 g	Chronic alcohol‐induced liver damage model (alcohol (50%, v/v, 15 mL/kg) was taken orally once daily for 8 weeks)	Water extracts of jujube fruits; 100, 200, and 400 mg/kg/day	NM	By intragastric, 4 weeks (the intervention was performed 4 weeks after the start of modeling)	SOD, MDA, GSH‐Px, body weight, liver index, TC, TG, ALT, AST, LDH, IL‐6, TNF‐α, iNOS, NF‐κB p65, liver pathology	Liver
Huang et al. (2017)	Balb/c mice (male; 18/6), 20–22 g	Acute liver injury model (giving an intraperitoneal injection of a sublethal dose of APAP (350 mg/kg))	Ethanol extracts of jujube fruits (jujube flavonoids); 100, 200, and 400 mg/kg/day	0.3% (w/v) sodium carboxy methyl cellulose	By intragastric, 10 days (the intervention was performed before modeling)	SOD, GSH‐Px, MDA, GSH, ALT, AST, ALP, TB, TNF‐α, IL‐6, IL‐1β, NO, NF‐κB p65, Nrf2, NQO1, liver pathology	Liver
Xie et al. (2018)	ICR mice (male; 24/12), 20–24 g	Diabetes model (intrabitoneally injected citric acid–sodium citrate buffer containing STZ 0.2 mL/10 g, the concentration of streptozotocin was 200 mg/kg. After 7 days, the fasting blood glucose was greater than 11.1 mmol/L, indicating successful modeling)	Water extracts of jujube fruits (jujube polysaccharide); 400 and 800 mg/kg/day	NS	By intragastric, 28 days	SOD, MDA, TC, TG, body weight, fasting blood glucose, insulin, liver pathology, pancreas pathology	Serum
Cai et al. (2018)	Kunming mice (male; 36/12), 18–22 g	Alcoholic liver injury model (30% ethanol (liquor Erguotou) was administered intragastrically for 4 weeks)	Ethanol extracts of jujube fruits (triterpene acid); 25, 50, and 100 mg/kg/day	Equivalent solvent (0.5% sodium carboxymethylcellulose solution)	By intragastric, 6 weeks (the modeling was performed from the 3rd week after intervention)	MDA, GSH‐Px, SOD, body weight, liver index, liver pathology, AST, TG, TC, HDL‐C, LDL‐C	Liver
Feng et al. (2019)	ICR mice (male; 30/10), 18–20 g	Diabetes model (200 mg/kg STZ was injected intraperitoneally. After a week, mice with blood sugar levels greater than 11.1 mmol/dL were considered successful)	Water extracts of jujube fruits; 100, 400, and 800 mg/kg/day	Same volume of NS	By intragastric, 4 weeks	SOD, GSH‐Px, MDA, bodyweight, insulin, fasting blood glucose, organ index of liver, spleen, and kidney, liver pathology, kidney pathology, pancreas pathology	Liver
Resim et al. (2020)(A)	SD rats (male; 12/6), 245–300 g	Sham operation model (the surgery was performed through a midline incision, and the cavernous nerves were identified on both sides without any additional maneuver)	Water extracts of jujube fruits; 200 and 400 mg/kg/day	NM	By oral, 2 weeks	MDA, CAT, SOD, prolidase, TGF‐β1, collagen types 1, collagen types 3, fibronectin, α Actin, β Actin, corpora cavernosa pathology	Serum
Resim et al. (2020)(B)	SD rats (male; 12/6), 245–300 g	Cavernous nerve injury model (cavernosal nerves on both sides were surgically dissected and squeezed with clamps)	Water extracts of jujube fruits; 200 and 400 mg/kg/day	Distilled water	By oral, 2 weeks	MDA, CAT, SOD, prolidase, TGF‐β1, collagen types 1, collagen types 3, fibronectin, α Actin, β Actin, corpora cavernosa pathology	Serum
Mohebbati et al. (2021)(A)	Wistar rats (male; 5/5), 230–270 g	None	Ethanol extracts of jujube fruits; 200 mg/kg/day	NS	By oral, 3 weeks	MDA, SOD, CAT, total thiol content, sperm count, sperm motility, abnormal spermatozoa (abnormal head), abnormal spermatozoa (abnormal tail), testosterone, luteinizing hormone, follicle‐stimulating hormone, testis weight, testis pathology	Testis
Mohebbati et al. (2021)(B)	Wistar rats (male; 5/5), 230–270 g	Testicular toxicity model (adriamycin with a single dose (10 mg/kg, iv) was injected into the vein of the tail)	Ethanol extracts of jujube fruits; 200 mg/kg/day	NM	By oral, 3 weeks	MDA, SOD, CAT, total thiol content, sperm count, sperm motility, abnormal spermatozoa (abnormal head), abnormal spermatozoa (abnormal tail), testosterone, luteinizing hormone, follicle‐stimulating hormone, testis weight, testis pathology	Testis

Abbreviations: ALP, alkaline phosphatase; ALT, alanine aminotransferase; APAP, acetaminophen; AST, aspartate transaminase; BUN, blood urea nitrogen; CAT, catalase; CCl4, tetrachloromethane; CK, creatine kinase; GOT, glutamic oxalacetic transaminase; GPT, glutamic‐pyruvic transaminase; GSH, glutathione; GSH‐Px, glutathione peroxidase; HDL‐C, high‐density lipoprotein cholesterol; HPMC, hydroxypropyl methylcellulose; IL‐10, interleukin‐10; IL‐1β, Interleukin‐1 beta; IL‐2, interleukin‐2; IL‐4, interleukin‐4; IL‐6, Interleukin‐6; LDH, lactate dehydrogenase; LDL‐C, low‐density lipoprotein cholesterol; MDA, malonaldehyde; MWM, Morris water maze; NK cell, natural killer cell; NM, not mentioned; NO, nitric oxide; NQO1, quinone oxidoreductase 1; Nrf2, nuclear factor erythroid 2‐related factor 2; NS, normal saline; OFT, open‐field test; PVP K30, polyvinylpyrrolidone; SD, Sprague–Dawley; SIJ, single intraperitoneal injection; SOD, superoxide dismutase; STZ, streptozotocin; TB, total bilirubin; TC, total cholesterol; TG, triglyceride; TGF‐β1, transforming growth factor‐beta‐1; TNF‐α, tumor necrosis factor‐α; T‐SOD, total superoxide dismutase; TST, tail suspension test.

### 3.3 Risk of bias and quality of included studies

The risk of bias scores of all studies ranged from 2 to 4, with one study obtaining two points, nine studies scoring three points, and nine studies scoring four points. Among the 19 studies included, no study described the methods used to generate the allocation sequence, and three studies did not mention the method of randomization (Huang et al., 2017; Resim et al., 2020; Shen et al., 2009). Four studies reported that the baseline characteristics were similar between groups (Feng et al., 2019; Li et al., 2005; Sheng, 2004; Xie et al., 2018). It was not made clear by any of the research whether or not the allocation to the various groups was sufficiently concealed. Seven studies were considered with random housing because the experimental animals were housed in the same environment and put on a free diet (Cai et al., 2018; Chi et al., 2015; Du & Liu, 2008; Huang et al., 2017; Shen et al., 2009; Wang, 2010; Wang et al., 2012). Adequate information about the blinding of caregivers or investigators was not offered by any of the studies. None of the studies could determine the exact risk of randomization and blinding of outcome evaluation. One study was evaluated to provide incomplete outcome data (Resim et al., 2020), and the primary outcome data of other studies were complete. The results of the two studies were inconsistent with the description provided in the study methods; hence, these studies could have reported selective results, which could be considered high risk (Feng et al., 2019; Xie et al., 2018). No other source deviation was observed in the studies. The details are provided in Table 2.

**TABLE 2:** The methodological quality of the included studies.

Study	A	B	C	D	E	F	G	H	I	J	Total
Sheng (2004)	?	+	?	?	?	−	?	+	+	+	4
Li et al. (2005)	?	+	?	?	?	−	?	+	+	+	4
Gu et al. (2006)	?	?	?	?	?	−	?	+	+	+	3
Du and Liu (2008)	?	?	?	+	?	−	?	+	+	+	4
Shen et al. (2009)	−	?	?	+	?	−	?	+	+	+	4
Wang (2010)	?	?	?	+	?	−	?	+	+	+	4
Kang and Li (2010)	?	?	?	?	?	−	?	+	+	+	3
Wang et al. (2012)	?	?	?	+	?	−	?	+	+	+	4
Gu et al. (2012)	?	?	?	?	?	−	?	+	+	+	3
Shao and Tang (2015)	?	?	?	?	?	−	?	+	+	+	3
Chi et al. (2015)	?	?	?	+	?	−	?	+	+	+	4
Liu et al. (2015)	?	?	?	?	?	−	?	+	+	+	3
Liu et al. (2017)	?	?	?	?	?	−	?	+	+	+	3
Huang et al. (2017)	−	?	?	+	?	−	?	+	+	+	4
Xie et al. (2018)	?	+	?	?	?	−	?	+	−	+	3
Cai et al. (2018)	?	?	?	+	?	−	?	+	+	+	4
Feng et al. (2019)	?	+	?	?	?	−	?	+	−	+	3
Resim et al. (2020)	−	?	?	?	?	−	?	−	+	+	2
Mohebbati et al. (2021)	?	?	?	?	?	−	?	+	+	+	3

*Note*: Selection bias: A, Sequence generation; B, Baseline characteristics; C, Allocation concealment. Performance bias: D, Random housing; E, Blinding. Detection bias: F, Random outcome assessment; G, Blinding. Attrition bias: H, Incomplete outcome data. Reporting bias: I, Selective outcome reporting. Other: J, Other sources of bias. ?, unclear; +, low risk; −, high risk.

### 3.4 Effects on SOD

**FIGURE 3:**
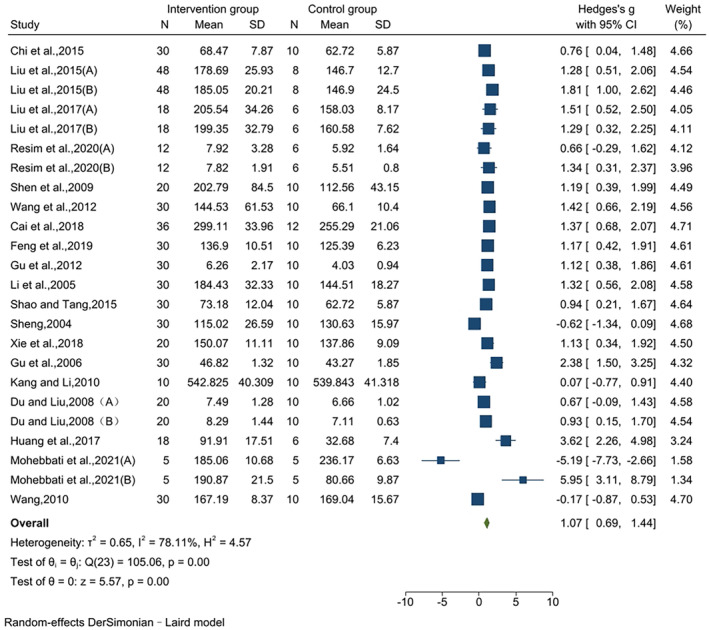
Effects on SOD.

The effect of jujube fruit extracts on SOD levels was reported in 24 pairwise comparisons using data from 19 studies. The combined findings indicated that in comparison to that in the control group, the SOD level in the intervention group was considerably higher, and the effect value was large (SMD = 1.07, 95% CI [0.69, 1.44], *p* < .01). The results of the heterogeneity test indicated significant heterogeneity (*I* ^2^ = 78.11%, *p* < .01) (Figure 3).

### 3.5 Effects on MDA

**FIGURE 4:**
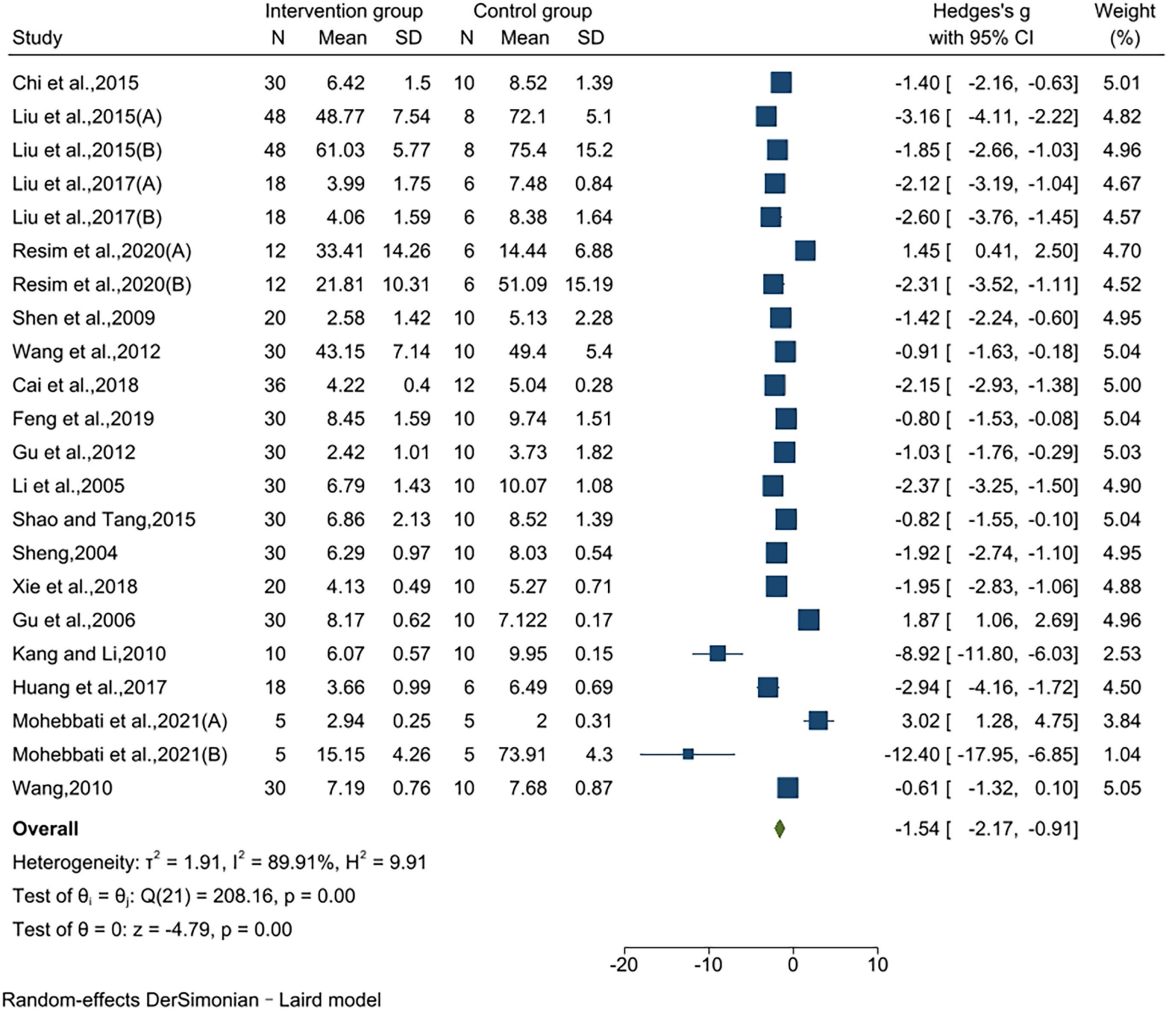
Effects on MDA.

The effect of jujube fruit extracts on MDA levels was reported in 22 pairwise comparisons using data from 18 studies. In the intervention group, the level of MDA was considerably lower than that in the control group, and the effect value was large (SMD = −1.54, 95% CI [−2.17,‐0.91], *p* < .01). The results of the heterogeneity test indicated significant heterogeneity (*I* ^2^ = 89.91%, *p* < .01) (Figure 4).

### 3.6 Effects on GSH‐Px

**FIGURE 5:**
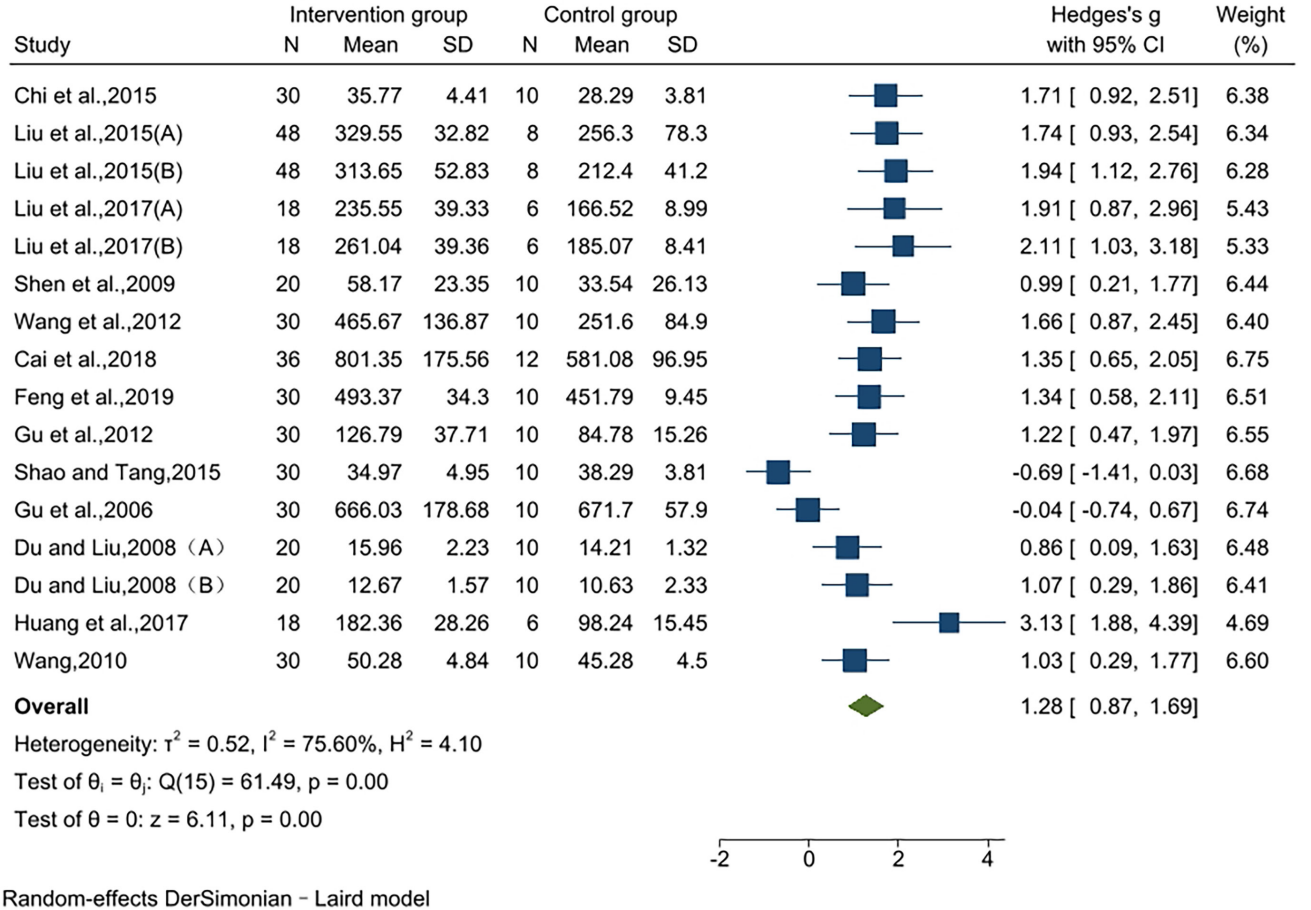
Effects on GSH‐Px.

The effect of jujube fruit extracts on GSH‐Px was reported in 16 pairwise comparisons using data from 13 studies. Compared to that in the control group, the GSH‐Px level was significantly higher in the intervention group, and the effect value was large (SMD = 1.28, 95% CI [0.87, 1.69], *p* < .01). The results of the heterogeneity test indicated significant heterogeneity (*I* ^2^ = 75.60%, *p* < .01) (Figure 5).

### 3.7 Effects on CAT

**FIGURE 6:**
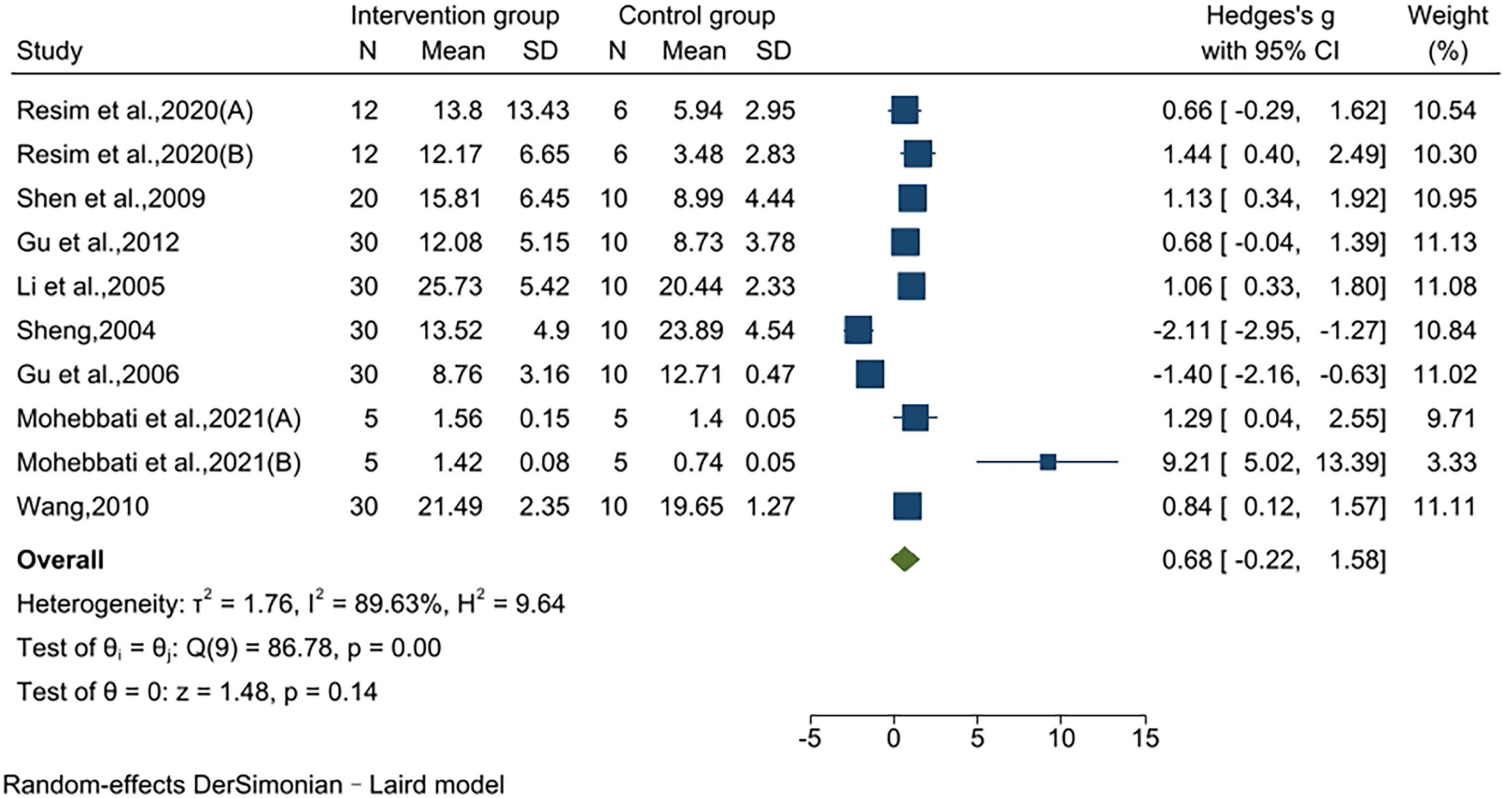
Effects on CAT.

The effect of jujube fruit extracts on CAT was reported in 10 pairwise comparisons using data from eight studies. No statistically significant difference was observed between the intervention and control groups concerning the effects on CAT (SMD = 0.68, 95% CI [−0.22, 1.58], *p* = .14). Results of the heterogeneity test indicated significant heterogeneity (*I* ^2^ = 89.63%, *p* < .01) (Figure 6).

### 3.8 Subgroup analysis

The levels of SOD, MDA, GSH‐Px, and CAT showed high heterogeneity. To investigate the source of heterogeneity, we performed subgroup analyses on animal models, type of extracts, duration of investigation, and source of target parameters (Table 3).

**TABLE 3:** Subgroup analyses in SOD, MDA, GSH‐Px, and CAT with the ex ante parameters.

Comparison	Subgroup	Number	SMD [95% CI]	*p* for meta‐analysis	*I* ^2^ (%)	*p* for heterogeneity
SOD						
Animal model	Injury model	14	0.887 [0.627, 1.147]	<.001	31.96	.12
	Toxicity model	7	2.050 [1.354, 2.746]	<.001	72.32	.001
	No model	3	‐1.105 [−3.410, 1.201]	.348	93.47	<.001
Type of extracts	Water extracts	13	1.115 [0.790, 1.439]	<.001	51.41	.016
	Ethanol extracts	9	1.043 [0.031, 2.055]	.043	89.87	<.001
	Not mentioned	2	0.796 [0.255, 1.338]	.004	0	.644
Duration	Long	12	0.943 [0.612, 1.274]	<.001	54.47	.012
	Medium	10	1.125 [0.190, 2.060]	.018	88.18	<.001
	Short	2	1.533 [0.972, 2.093]	<.001	0	.358
Source of target parameters	Serum	9	0.969 [0.578, 1.361]	<.001	51.11	.037
	Liver	9	1.496 [1.153, 1.839]	<.001	32.57	.157
	Others	6	0.337 [−0.955, 1.629]	.609	90.56	<.001
MDA						
Animal model	Injury model	12	−1.569 [−2.265, −0.873]	<.001	85.79	<.001
	Toxicity model	7	−1.948 [−3.462, −0.435]	.012	93.90	<.001
	No model	3	−0.572 [−2.958, 1.813]	.638	93.52	<.001
Type of extracts	Water extracts	13	−1.023 [−1.707, −0.340]	.003	88.17	<.001
	Ethanol extracts	9	−2.521 [−3.700, −1.342]	<.001	89.74	<.001
Duration	Long	10	−1.651 [−2.060, −1.243]	<.001	58.29	.01
	Medium	10	−1.431 [−2.819, −0.043]	.043	93.68	<.001
	Short	2	−2.483 [−3.774, ‐1.191]	<.001	76.79	.038
Source of target parameters	Serum	7	−1.366 [−2.887, 0.155]	.078	94.20	<.001
	Liver	9	−1.926 [−2.482, −1.370]	<.001	71.67	<.001
	Others	6	−1.275 [−2.630, 0.081]	.065	90.23	<.001
GSH‐Px						
Animal model	Injury model	10	1.153 [0.668, 1.639]	<.001	73.00	<.001
	Toxicity model	6	1.502 [0.713, 2.291]	<.001	81.44	<.001
Type of extracts	Water extracts	9	1.103 [0.480, 1.726]	.001	81.65	<.001
	Ethanol extracts	5	1.711 [1.135, 2.287]	<.001	57.14	.053
	Not mentioned	2	0.964 [0.413, 1.514]	.001	0	.702
Duration	Long	9	1.172 [0.625, 1.720]	<.001	75.98	<.001
	Medium	5	1.265 [0.403, 2.127]	.004	81.86	<.001
	Short	2	1.836 [1.261, 2.410]	<.001	0	.728
Source of target parameters	Serum	5	0.571 [−0.264, 1.407]	.18	83.77	<.001
	Liver	9	1.674 [1.339, 2.009]	<.001	26.68	.207
	Others	2	1.122 [0.597, 1.648]	<.001	0	.723
CAT						
Animal model	Injury model	4	0.847 [0.434, 1.261]	<.001	0.00	.658
	Toxicity model	3	1.925 [−1.008, 4.857]	.198	94.90	<.001
	No model	3	0.064 [−2.187, 2.315]	.956	94.48	<.001
Type of extracts	Water extracts	6	0.531 [−0.280, 1.342]	.199	83.42	<.001
	Ethanol extracts	4	1.565 [−0.959, 4.089]	.224	94.51	<.001
Duration	Long	3	−0.112 [−1.971, 1.747]	.906	94.38	<.001
	Medium	7	1.058 [−0.026, 2.142]	.056	87.54	<.001
Source of target parameters	Serum	3	0.209 [−1.542, 1.960]	.815	90.85	<.001
	Liver	1	1.129 [0.337, 1.921]	.005	/	/
	Others	6	0.941 [−0.391, 2.272]	.166	91.43	<.001

#### 3.8.1 Animal model

We categorized the animal models into three subgroups, namely the injury model (including the chronic fatigue syndrome model, alcoholic liver model, diabetes model, hyperlipidemia model, exercise‐induced fatigue model, and surgical model), toxicity model (including the toxic liver injury model and testicular toxicity model), and unmodeled. The results of the subgroup analysis showed that the effects of the animal model on SOD, MDA, GSH‐Px, and CAT were similar. The effect size of the toxicity model was better than that of the injury model, which was better than that of the unmodeled ones. However, only the findings for SOD had statistical significance (SMD 2.050 vs. SMD 0.887 vs. SMD − 1.105, *p* = .002). The heterogeneity of MDA and GSH‐Px was significant, whereas the heterogeneity of SOD levels (*I* ^2^ = 31.96%, *p* = .120) and CAT (*I* ^2^ = 0%, *p* = .658) in the injury model group was significantly lower. This suggests that differences in the animal model may be a source of high heterogeneity in the SOD and CAT levels.

#### 3.8.2 Type of extracts

Based on whether the medium of maceration was water or ethanol, the types of extracts were roughly divided into two groups: water extracts and ethanol extracts. The findings of the subgroup analysis revealed that for MDA, GSH‐Px, and CAT, the effect size of the ethanol extracts of jujube fruits was considerably better than that of water extracts, and only the findings for MDA had statistical significance (SMD −2.521 vs. SMD −1.023, *p* = .031). However, for SOD, the effect size of the ethanol extract was slightly lower than that of the water extract, and the difference between the two groups was not statistically significant. The heterogeneity in the water extracts group was lower for SOD (*I* ^2^ = 51.41%, *p* = .016), and the heterogeneity in the ethanol extract group was lower for GSH‐Px (*I* ^2^ = 57.14%, *p* = .053). The heterogeneity of the results may be partly attributed to the type of extracts.

#### 3.8.3 Duration

We divided the duration of intervention into three subgroups: long (>3 weeks), medium (1–3 weeks), and short (<1 week). The results of the subgroup analysis indicated that any duration of intervention with the jujube fruit extracts exerted positive effects on the SOD, MDA, and GSH‐Px levels, whereas the long treatment yielded minor negative effects on the CAT levels. The subgroup analysis results of SOD, MDA, GSH‐Px, and CAT were not statistically significant. The heterogeneity in the long treatment group of SOD (*I* ^2^ = 54.47%, *p* = .012) and MDA (*I* ^2^ = 58.29%, *p* = .010) was lower, suggesting that the duration of treatment may have partly influenced the heterogeneity.

#### 3.8.4 Source of target parameters

We divided the source of target parameters into three subgroups: serum, liver, and others (including testes and multiple sources). The results of the subgroup analysis showed that the liver group had the largest effect size on the SOD, MDA, GSH‐Px, and CAT levels. Concerning GSH‐Px, the liver group showed better results than the other group, which showed better results than the serum group; the differences were statistically significant (SMD 1.675 vs. SMD 1.122 vs. SMD 0.571, *p* = .025). The heterogeneity of SOD in the serum group was low (*I* ^2^ = 52.11%, *p* = .037). The heterogeneity of SOD (*I* ^2^ = 32.57%, *p* = .157) and GSH‐Px (*I* ^2^ = 26.68%, *p* = .207) in the liver group was significantly lower, suggesting that the source of the target parameters could partially explain the heterogeneity of the study data.

### 3.9 Sensitivity analysis and publication bias

We examined the sensitivity of SOD, MDA, GSH‐Px, and CAT. The results of a leave‐one‐out sensitivity analysis revealed that the effect sizes were reliable and unaffected by any particular study.

**FIGURE 7:**
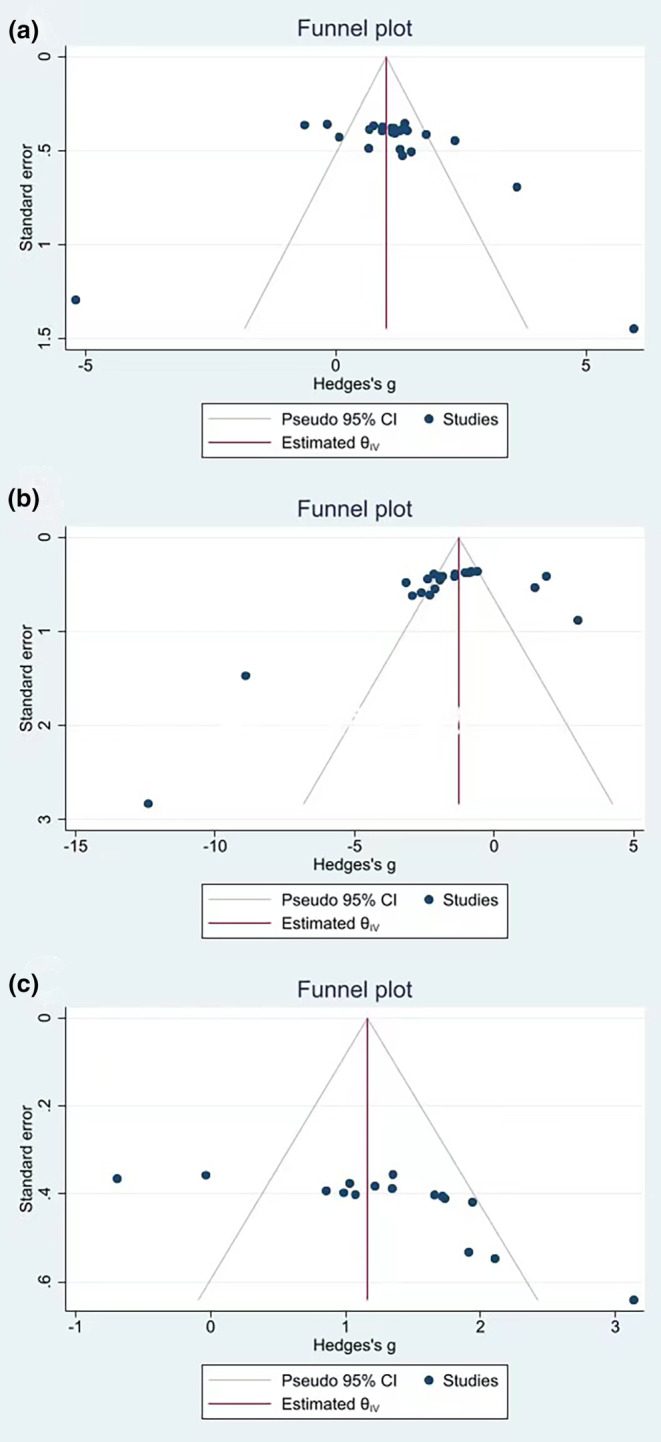
Egger's publication bias plot for SOD, MDA, and GSH‐Px.

Since the number of included studies was greater than 10, we evaluated the publication bias of SOD, MDA, and GSH‐Px (Figure 7). The Egger publication bias plot showed that the findings for SOD (*p* = .6831) had no significant publication bias, whereas those for MDA (*p* = .0004) and GSH‐Px (*p* = .0032) did.

## 4 DISCUSSION

The purpose of this systematic review and meta‐analysis was to assess the effects of jujube fruit extracts on oxidative stress levels in rodent models. Nineteen studies were included. Jujube fruit extracts could significantly decrease MDA levels and increase SOD and GSH‐Px levels but exerted no significant effect on CAT levels. In this meta‐analysis, the results of these outcome indicators showed a high degree of heterogeneity. According to the results of the subgroup analysis, the animal model was found to be the potential source of heterogeneity for SOD. The type of extracts was identified as the potential source of heterogeneity for MDA. The target parameter source was considered as the potential source of heterogeneity for GSH‐Px.

An increasing body of research suggests that oxidative stress plays a major role in the occurrence and progression of multiple diseases and that controlling oxidative stress can significantly enhance biological metabolism and slow down the progression of diseases. Biomarkers are crucial for assessing the progression of oxidative stress and elucidating the pathogenic mechanisms of the disease. Among the antioxidant defense systems, SOD, CAT, and GSH‐Px are the best‐known biomarkers and major contributors to protection against oxidative damage (Demirci‐Çekiç et al., 2022; Lichtenberg et al., 2023; Marrocco et al., 2017). SOD, which catalyzes the conversion of superoxide radicals into oxygen and hydrogen peroxide, is the initial line of defense against ROS damage (Zhao et al., 2021). Hydrogen peroxide is a substrate for CAT and GSH‐Px, whereas CAT is the core antioxidant enzyme in most organisms that catalyzes the decomposition of hydrogen peroxide into water and oxygen (Baker et al., 2023), and GSH‐Px catalyzes the reduction of hydrogen peroxide or organic hydroperoxides to water or the corresponding alcohol using the appropriate reducing agent (Pei et al., 2023). These enzymatic antioxidants work collectively to reduce ROS levels and limit their toxicity. Lipids are highly susceptible to oxidative stress, and MDA, as a comparatively stable metabolite during lipid peroxidation between ROS and polyunsaturated fatty acids, is considered to be a typical biomarker of lipid peroxidation. Changes in MDA can reflect the degree of oxidative stress; hence, this molecule is commonly used as a measure of oxidative stress (Li, Muhammad, et al., 2023; Li, Pan, et al., 2023; Mas‐Bargues et al., 2021). Jujube fruit extracts can reduce the levels of MDA and increase the levels of SOD and GSH‐Px, indirectly confirming that they can reduce the levels of lipid peroxidation and activate the inherent antioxidative enzyme system to balance the oxidative stress status in multiple tissues and organs. However, jujube fruit extracts did not significantly raise CAT levels in the included studies. Possibly, the heterogeneity of the included literature and its limited scope prevented jujube fruit extracts from playing a larger role in this regard. It is worth mentioning that jujube is rich in nonenzymatic plant compounds such as vitamins (especially vitamin C), polyphenols, and triterpenoids (Lu et al., 2021), and while these nonenzymatic antioxidants may not be able to outcompete enzymatic antioxidants that can catalyze the depletion of ROS, they are equally indispensable (Meulmeester et al., 2022).

The findings of our subgroup analysis suggested that the animal model may influence the antioxidant effect. When the researchers used toxicity models, jujube fruit extracts exhibited a better effect on oxidative stress levels, perhaps because the mechanism of action of oxidative stress is different in different diseases. In toxic diseases, oxidative stress frequently plays a significant role because toxins trigger excessive ROS production, which results in oxidative damage (McGill & Hinson, 2020; Unsal et al., 2021). In addition to this, oxidative stress is merely one of the many factors that influence multiple diseases, and it develops after other causes have already triggered the pathology. Oxidative stress interferes with many signaling pathways, thereby affecting various biological processes and aggravating the symptoms of diseases by modifying proteins, fostering inflammation, triggering apoptosis, and deregulating autophagy, among other mechanisms (Forman & Zhang, 2021). It is worth mentioning that exercise is one of the common physiological conditions linked to increased oxidative stress, and high‐intensity exercise leads to an overproduction of ROS and oxidative damage to muscle fibers (Arazi et al., 2021).

The type of jujube extract also affects the antioxidant effect. People extract the desired natural active ingredients from jujube through a series of processes, in which jujube polysaccharide is the primary research object (Wang, 2021). Notably, both crude extracts of jujube obtained by simple extraction and polysaccharides, triterpene acid, and flavonoids obtained by complex isolation and purification processes have good antioxidant effects. At present, the primary medium used for extracting active ingredients from jujube is water or ethanol. Owing to the different polarities and solubilities of the target bioactive ingredients, appropriate extraction methods must be used. For extracting polar molecules, water is the most cost‐effective and safest solvent, and it also functions effectively. However, when the goal is to derive a compound with less polarity, an organic solvent like ethanol works better (Plaskova & Mlcek, 2023). The yield, content, and antioxidant activity of the extracts were significantly influenced by the extraction technique and different processing factors. Owing to the significant differences in the processes and technologies used in the included literature, we did not investigate the extraction methods and the components of the extracts obtained. However, we roughly categorized the extracts into water extracts and ethanol extracts based on the selected maceration medium. The outcomes of the subgroup analysis demonstrated that the effect of water extracts on improving oxidative stress in SOD was slightly better than that of ethanol extracts. However, the effect of ethanol extracts on MDA, GSH‐Px, and CAT was considerably better than that of water extracts, which may indicate that the substances soluble in ethanol in jujube can better regulate the levels of oxidative stress. However, these findings need further validation from more relevant studies.

The antioxidant effect of jujube extracts could be exerted on blood and different tissues and organs, and its effect on the liver is most significant. All data support this result. The human body is a unified whole, with various tissues and organs interacting with each other and each having its unique properties. A crucial metabolic organ, the liver has an abnormal buildup of lipids, metabolic products, and toxins that can disrupt the redox state and harm cells (Sadasivam et al., 2022). This implies that the liver is susceptible to oxidative stress and is also an important target for drug action. Blood travels through the body, exchanging substances with various organs and tissues. The oxidative stress index in serum can reflect the overall oxidative stress state to a certain extent. The outcomes of the subgroup analyses suggested that jujube fruit extracts were effective in regulating systemic oxidative stress. Although these extracts demonstrated particularly strong efficacy in the liver, this notable effect may be attributed to the inherent characteristics of the liver itself. Researchers have primarily focused on the liver to determine oxidative stress levels, and there is insufficient data on other tissues and organs. This knowledge gap is worthy of further exploration.

In the included studies, the timing of the therapeutic intervention is varied. However, regardless of whether the jujube fruit extracts were used as a preventive drug or directly to treat disease, the results indicated the good antioxidant capacity of jujube fruit extracts. In many of the included studies, the dosage of jujube extracts was categorized into different levels. The results of most of these studies indicated that the dose effect of jujube extracts showed a curve in which the antioxidant effect of medium doses was superior to that of high doses, which in turn was superior to that of low doses. It tentatively demonstrated a dose–effect relationship between jujube fruit extracts and their antioxidant capacity. Owing to the substantial differences in dose distribution in the included studies, we combined all doses in the study, which, to some extent, weakened the therapeutic effect of jujube fruit extracts.

Multiple dietary supplements are touted as antioxidants, but only a few have been shown to promote health benefits. Similarly, multiple plant extracts exhibit antioxidant activity, but not all of them can be used as dietary supplements (Ali et al., 2020). Quality, safety, and efficacy are important factors of consideration for dietary supplements (Dwyer et al., 2018; Féart, 2020).

Keeping quality control‐related factors aside, the efficacy of the jujube fruit has been demonstrated in clinical applications, where it is often used to treat endocrine, cardiovascular, psychiatric, gastrointestinal, and respiratory disorders (Wang, 2021). However, as with most botanical dietary supplements, the type and amount of evidence for the efficacy of jujube fruit is limited, and the vast majority of evidence now focuses on the antioxidant effects of jujube fruit. We combined different doses of jujube fruit extracts and performed a meta‐analysis while underestimating the antioxidant effect. The results indicated that the extracts could still significantly modulate the levels of SOD, MDA, and GSH‐Px, and the effect values were all large, which confirms the effectiveness of the jujube fruit extracts in resisting oxidative stress. Jujube is one of the most commonly used herbs in TCM treatments as well as a fruit that can be consumed daily, and its extensive use over centuries indicates its safety. No study has reported the side effects of jujube. Jujube is susceptible to infestations by fungi such as *Aspergillus niger* during growth and ripening. However, even though the quality of the jujube fruit can be affected by *A. niger*, owing to the characteristics of the fruit, it does not accumulate ochratoxin A, a toxic secondary fungal metabolite that is widespread in food and agricultural products (Xin et al., 2023). Based on these characteristics, we can determine that jujube is an excellent antioxidant dietary supplement. In addition, the jujube fruit has a high yield, good flavor, and reasonable price and is easy to store. Of the many antioxidant dietary supplements available, it may not be the most potent in terms of its antioxidant capacity, but it is certainly one of the healthiest and most easily accessible fruits available to the general public.

This study had several limitations. First, the number of studies in which the effects of jujube fruit extracts on oxidative stress were evaluated was small. Second, the majority of the included studies had poor quality, and there were substantial interstudy differences and high heterogeneity of results. Finally, a publication bias for MDA and GSH‐Px was observed, which might have caused the benefits of jujube fruit extracts to be overestimated.

## 5 CONCLUSION

Jujube fruit extracts are healthy and effective antioxidant dietary supplements with beneficial effects on SOD, GSH‐Px, and MDA levels. These extracts may be an effective adjunctive therapy for diseases in which oxidative stress is an important pathological factor. However, the overall methodological quality of the included studies was low, and large‐scale, long‐term, and high‐quality animal modeling trials and human clinical trials are still needed to further explore and demonstrate the antioxidant properties of jujube fruit extracts.

## AUTHOR CONTRIBUTIONS

**Di Zhu:** Conceptualization (equal); data curation (equal); formal analysis (equal); methodology (equal); writing – original draft (equal). **Yu Zhu:** Conceptualization (supporting); methodology (supporting); writing – review and editing (supporting). **Hao Tan:** Conceptualization (supporting); visualization (equal); writing – review and editing (supporting). **Rui Ding:** Conceptualization (supporting); supervision (equal); writing – review and editing (supporting). **Qiangqiang Dai:** Conceptualization (supporting); writing – review and editing (supporting). **Xiaoming Du:** Conceptualization (supporting); writing – review and editing (supporting). **Yulin Liu:** Conceptualization (supporting); writing – review and editing (supporting). **Rensong Yue:** Supervision (equal); writing – review and editing (supporting).

## FUNDING INFORMATION

This work was supported by grants from the Science and Technology Research Special project of the Sichuan Provincial Administration of Traditional Chinese Medicine (No. 2021ZD011).

## CONFLICT OF INTEREST STATEMENT

The authors declare that they have no conflict of interest.

## Supporting information


Table S1

## Data Availability

We confirm that the data supporting the findings of this study are available within the article.
