# Supplementary material for: Effects of jujube (Ziziphus jujuba mill.) fruit extracts on oxidative stress: A systematic review and meta‐analysis of rodent studies
Source: Food Sci Nutr. 2024 May 22;12(8):5312–28. doi: 10.1002/fsn3.4234 (PMC11317725; doi:10.1002/fsn3.4234)
Supplement: Supplementary file 1 — Table S1 [file FSN3-12-5312-s001.docx]

Table S1. Search strategy

Search Strategy for PubMed

| Number | Search terms |
| --- | --- |
| 1 | "Ziziphus"[Mesh Terms] |
| 2 | "Ziziphus"[Title/Abstract] OR "Jujube"[Title/Abstract] OR "Chinese jujube"[Title/Abstract] OR "Ziziphus jujuba Mill"[Title/Abstract] OR "jujube polysaccharide"[Title/Abstract] |
| 3 | 1 OR 2 |
| 4 | "inflammation"[Mesh Terms] OR "cytokines"[Mesh Terms] OR "[Interleukins](https://www.ncbi.nlm.nih.gov/mesh/68007378)"[Mesh Terms] OR "Tumor Necrosis Factor-alpha"[Mesh Terms] OR "C-Reactive Protein"[Mesh Terms] |
| 5 | "inflammation"[Title/Abstract] OR "inflammatory"[Title/Abstract] OR "cytokines"[Title/Abstract] OR "[Interleukin](https://www.ncbi.nlm.nih.gov/mesh/68007378)"[Title/Abstract] OR "Tumor Necrosis Factor-alpha"[Title/Abstract] OR "C-Reactive Protein"[Title/Abstract] |
| 6 | 4 OR 5 |
| 7 | "Oxidative Stress"[Mesh Terms] OR "Antioxidants"[Mesh Terms] OR "Superoxide Dismutase"[Mesh Terms] OR "Glutathione"[Mesh Terms] OR "Glutathione Peroxidase"[Mesh Terms] OR "Malondialdehyde"[Mesh Terms] OR "Catalase"[Mesh Terms] |
| 8 | "Oxidative Stress"[Title/Abstract] OR "antioxidant"[Title/Abstract] OR "superoxide dismutase"[Title/Abstract] OR "glutathione "[Title/Abstract] OR "glutathione peroxidase"[Title/Abstract] OR "malondialdehyde"[Title/Abstract] OR "catalase"[Title/Abstract] |
| 9 | 7 OR 8 |
| 10 | 6 OR 9 |
| 11 | "rats"[Mesh Terms] OR "mice"[Mesh Terms] OR "animals"[Mesh Terms] |
| 12 | "rat"[Title/Abstract] OR "mice"[Title/Abstract] OR "mouse"[Title/Abstract] OR "animal"[Title/Abstract] OR "rodent"[Title/Abstract] OR "murinae"[Title/Abstract] |
| 13 | 11 OR 12 |
| 14 | 3 AND 10 AND 13 |

Search Strategy for Web of Science

| Number | Search terms |
| --- | --- |
| #1 | TS=（"Ziziphus"） OR TS=("Jujube") OR TS=("Chinese jujube") OR TS=("Ziziphus jujuba Mill") OR TS=("jujube polysaccharide"） |
| #2 | TS=（"inflammation"） OR TS=("inflammatory") OR TS=("cytokines") OR TS=("Interleukin") OR TS=("Interleukins") OR TS=("Tumor Necrosis Factor-alpha") OR TS=("C-Reactive Protein"） OR TS=("Oxidative Stress") OR TS=("Oxidative Stress biomarkers") OR TS=("antioxidant") OR TS=（"superoxide dismutase"） OR TS=("glutathione") OR TS=(glutathione peroxidase) OR TS=（"malondialdehyde") OR TS=("catalase") |
| #3 | TS=（"rat"） OR TS=("rats") OR TS=("mice") OR TS=("mouse") OR TS=("animal") OR TS=("animals"）OR TS=("rodent"）OR TS=("rodents"）OR TS=("murinae"） |
| #4 | ((#1) AND #2) AND #3 |

Search Strategy for Embase

| Number | Search terms |
| --- | --- |
| #1 | 'Ziziphus'/exp |
| #2 | 'Ziziphus':ab,ti OR 'Jujube':ab,ti OR 'Chinese jujube':ab,ti OR 'Ziziphus jujuba Mill':ab,ti OR 'jujube polysaccharide':ab,ti |
| #3 | #1 OR #2 |
| #4 | 'inflammation'/exp |
| #5 | 'inflam*':ab,ti OR 'cytokines':ab,ti OR 'Interleukin*':ab,ti OR 'Tumor Necrosis Factor-alpha':ab,ti OR 'C-Reactive Protein':ab,ti |
| #6 | #4 OR #5 |
| #7 | 'Oxidative Stress'/exp |
| #8 | 'Oxidative Stress':ab,ti OR 'antioxidant':ab,ti OR 'superoxide dismutase':ab,ti OR 'glutathione':ab,ti OR 'glutathione peroxidase':ab,ti OR 'malondialdehyde':ab,ti OR 'catalase':ab,ti |
| #9 | #7 OR #8 |
| #10 | #6 OR #9 |
| #11 | 'animals'/exp |
| #12 | 'rat*':ab,ti OR 'mice':ab,ti OR 'mouse':ab,ti OR 'animal*':ab,ti OR 'rodent*':ab,ti OR 'murinae':ab,ti |
| #13 | #11 OR #12 |
| #14 | #3 AND #10 AND #13 |
